# Subpial resection of epileptogenic tubers: operative nuances and lessons learned

**DOI:** 10.3171/2024.4.FOCVID2411

**Published:** 2024-07-01

**Authors:** Nisha Giridharan, Collin W. English, Marcus Wong, Howard L. Weiner

**Affiliations:** 1 Department of Neurosurgery, Baylor College of Medicine, Houston;; 2 Department of Neurosurgery, Houston Methodist Hospital, Houston; and; 3 Division of Pediatric Neurosurgery, Texas Children’s Hospital, Houston, Texas

**Keywords:** epilepsy surgery, subpial dissection, tuber, tuberous sclerosis

## Abstract

Tuberous sclerosis complex (TSC) is an autosomal dominant neurocutaneous disorder. Tubers of the central nervous system are a hallmark of the disorder and often cause epilepsy. Many TSC patients fail to achieve seizure control with medication alone. Several case series have demonstrated high seizure freedom rates after resective surgery. However, the technique for the resection of epileptogenic tubers has largely been unreported. Here the authors present 2 cases to illustrate their multistage approach for localizing and resecting the seizure onset zone in patients with TSC. At their institution, they have excellent seizure outcomes and a low complication rate with this technique.

The video can be found here: https://stream.cadmore.media/r10.3171/2024.4.FOCVID2411

## Transcript

In this video, we will outline our surgical decision-making for patients with tuberous sclerosis who have drug-resistant epilepsy. We will also describe our subpial technique for resecting epileptogenic tubers. Up to 75% of patients with tuberous sclerosis complex or TSC who have cortical tubers go on to develop drug-resistant epilepsy.^1,2^ Surgery has demonstrated efficacy for these patients. In fact, earlier control of seizures leads to better outcomes for cognitive, social, and behavioral development.^3^ In some patients, the primary epileptogenic source localizes to a single cortical tuber, which can be resected. However, in most cases the epileptogenic network is complex.^3–5^

### 0:59 Treatment Algorithm.

At our institution, all patients start with a phase I evaluation. If the data is suggestive of a localized seizure focus that is surgically accessible, we nowadays proceed to a craniotomy for resection of the seizure focus. These TSC patients are less common in our practice. If data from a phase I evaluation is suggestive of a multifocal seizure onset or nonlocalized source, then we bring the patient in for stereoelectroencephalography or sEEG lead placement.^6^ If the sEEG study points toward a large, solitary focus that is surgically accessible, we will proceed with a craniotomy for open resection. If there are multiple foci in a deep location that is smaller, we will opt for MRI-guided laser ablation. In many cases both options are reasonable, and the decision is made through detailed discussions with the family and our multidisciplinary epilepsy surgery team.^6^ This is the most common pathway in our practice because most of the patients that are referred to us are complex. Here we present 2 cases to illustrate our multistage approach for localizing and resecting the seizure onset zone in patients with TSC.

### 2:04 Case 1.

A 2-year-old left-handed female with TSC had two main seizure types. The first was focal impaired awareness seizures characterized by her eyes becoming glossy, motor arrest, and unresponsiveness. The second was gelastic seizures.

### 2:17 Preoperative Imaging.

MRI and CT images demonstrated a large constellation of partially calcified tubers in the right frontal region. Video-electroencephalography monitoring showed a right frontal ictal onset zone. After discussing her case in a multidisciplinary epilepsy surgery conference, we decided to perform a frontal lobectomy sparing the motor cortex and supplementary motor area or SMA. We decided to subsequently place subdural electrodes posteriorly over rolandic cortex and along the parietal lobe for postresection monitoring of motor and sensory regions. We rarely leave postoperative grids for chronic recording in our current practice. However, when we are more conservative in our resection due to proximity to eloquent cortex, we choose to leave a grid to monitor for the epileptogenicity of the remaining tissue and potentially for motor mapping. If the patient continues to have seizures after the initial resection, we discuss with the family about the risks and benefits of proceeding with a reresection.^5^

On the day of surgery, the patient was affixed in a Sugita head holder in the lateral position with the right side up. We drilled a large craniotomy exposing the entire right frontal lobe to facilitate both resection and placement of subdural grids. First, we mapped the boundaries of the tubers using stereotactic image guidance. Then we used phase reversal to map the primary motor cortex. Using the primary motor cortex as an anatomical landmark, we were also able to identify the SMA. It lies along the medial surface of the superior frontal gyrus anterior to the paracentral lobule and the posterior boundary is the precentral sulcus. Throughout the operation, we performed direct cortical stimulation of the motor strip to ensure motor signals were stable during the resection.

### 3:56 Surgical Procedure.

Next, we opened the pia over precentral gyrus marking our posterior boundary. Using a microdissector, we carefully retracted the tuber away from the pia of the precentral gyrus and the supplementary motor area. We reasoned that we could safely resect a tuber in this region to achieve a better seizure outcome without incurring an SMA syndrome. We spared the uninvolved, radiographically normal SMA to avoid deficits of contralateral weakness. The dissection continued circumferentially around to the anterior aspect of the frontal lobe and laterally and inferiorly to the sylvian fissure, capturing the entire frontal operculum.

### 4:30 Operative Technique.

We used a combination of bipolar cautery, suction, microdissectors, and an ultrasonic aspirator to resect and circumscribe the multiple tubers in the frontal lobe. We left the pia of the interhemispheric fissure intact and peeled off a large complex of calcified tubers in this area. The ultrasonic aspirator was then used to disconnect the frontal lobe from the underlying subcortical white matter. The tissue was sent for pathology.

### 4:55 Postoperative Course.

We placed a strip electrode over the motor cortex and a 64-contact grid over the parietal lobe centered on a large tuber in this location. No seizures were recorded, and we left the subdural electrodes in place for continued monitoring postoperatively. In the ICU, the patient went into electroclinical status epilepticus with left arm twitching and seizures arising from the right parietal grid contacts refractory to multiple antiepileptics. Imaging confirmed a gross-total resection of the right frontal tubers with no untoward effects. We suspected the seizures were related to irritation from the subdural electrodes given that there was no hemorrhage or other changes on postoperative imaging to explain her worsening seizures. Immediate removal of the subdural electrode grid led to seizure cessation, and the patient was discharged from the hospital a few days later. At her most recent follow-up 22 months after surgery, the patient has good control of her seizures on multiple antiepileptics. She is making progress with PT and walking some. She remains nonverbal but has improved receptive speech.

### 5:54 Case 2.

In this first case, we described an anatomical resection of lesions corresponding with the ictal onset zone. In this next case, we’ll show a patient who continued to have seizures despite focal laser ablations of tubers, and we opted for a more generous surgical resection. A 6-year-old boy with TSC and multiple tubers status post two stereoelectroencephalography-guided laser ablations presented with recurrent seizures, mostly at night. Given his two failed laser ablations and presurgical evaluation showing his seizures were coming from the right frontal region, a multidisciplinary epilepsy surgery team determined that a more generous right frontal lobectomy for resection of multiple tubers was the best treatment option.

### 6:34 Patient Positioning.

On the day of surgery, the patient was affixed in a Sugita head holder in the lateral position with the right side up. A curvilinear incision over the right frontal lobe was marked.

### 6:43 Pericranial Graft.

A pericranial graft was harvested and kept moist for dural closure later.

### 6:47 Craniotomy.

A large frontal craniotomy was drilled and the dura opened. There were some adhesions from the prior stereoelectroencephalography lead placement and ablations.

### 6:56 Monitoring.

A 4-contact strip was placed over the frontocentral region and phase reversal was performed to confirm the location of the primary motor cortex. We chose to use a grid and phase reversal for localization of the motor cortex so that we could leave the grid in place while we were performing the resection and check motor signals throughout to ensure stability.

### 7:14 Operative Technique.

We began the resection anterior to motor. The resection was performed using bipolar cautery and microscissors to incise the pia, and then a microdissector was used to dissect around the tubers in a subpial fashion. The areas that were ablated were extremely firm and scarred. We used bipolar cautery around these ablation corridors to remove them along with the tubers piecemeal. Once the lateral frontal region was resected, we then worked along the midline and interhemispheric region anterior to the supplementary motor area using the same technique. We removed the frontal polar and orbitofrontal tubers. Next, we worked laterally in the lateral orbitofrontal region and frontal operculum using the ultrasonic aspirator and microdissector in a subpial fashion until we completed the resection.

### 8:00 Specimen.

The tissue was sent for pathology.

### 8:03 Surgical Cavity.

And here is an image showing the postresection surgical cavity.

### 8:06 Postoperative Imaging.

Postoperative imaging showed an appropriate right frontal resection with a small epidural fluid collection but causing no mass effect. At 6 weeks postoperatively, the patient was dramatically better. Parents reported a significant improvement in seizures, much better behavior, cognition, comprehension, and happiness. However, by 9 months postoperative, the patient’s seizure frequency returned to about the same as preoperative. An EEG showed that the seizures are now predominantly originating from the left hemisphere. This was an extremely complex patient that had multiple laser ablations before we offered an open resection. This procedure was an attempt to palliate after a long discussion with the family and the right frontal area was the highest seizure burden preoperative. Now he continues to have seizures from other regions and the family is considering neuromodulation as a next treatment option.

### 8:51 Original Figure.

Open resection for the removal of tubers in TSC patients affords several advantages despite the rise of minimally invasive techniques.^7,8^ Surgeons can employ a subpial dissection technique to achieve a maximal safe resection while minimizing risks to surrounding critical structures. Tubers expand the involved gyrus and are pale compared to the surrounding tissue, as shown in figure A. Bipolar cautery and microscissors are used to open the pia over the lesion. Next, we utilize the Penfield 1 dissector and bipolar cautery to circumferentially dissect the tuber from the surrounding tissue shown in figures B and C. At the depth of the tuber, we amputate the lesion at the gray-white junction and inspect the underlying subcortical white matter for any remaining lesional tissue as shown in figure D. We send the specimen for pathology en bloc, if possible. Our institutional experience demonstrates excellent seizure outcomes and a low complication rate with this technique.^6^

## Disclosures

The authors report no conflict of interest concerning the materials or methods used in this study or the findings specified in this publication.

## Author Contributions

Primary surgeon: Weiner. Assistant surgeon: Wong. Editing and drafting the video and abstract: Giridharan, English, Wong. Critically revising the work: Weiner, Giridharan. Reviewed submitted version of the work: Weiner, Giridharan, English. Approved the final version of the work on behalf of all authors: Weiner. Supervision: Weiner.

## Supplemental Information

### Patient Informed Consent

The necessary patient informed consent was obtained in this study.

## References

[1] Curatolo P Moavero R de Vries PJ Neurological and neuropsychiatric aspects of tuberous sclerosis complex Lancet Neurol 2015 14 7 733 745 26067126 10.1016/S1474-4422(15)00069-1

[2] Thiele EA Managing and understanding epilepsy in tuberous sclerosis complex Epilepsia 2010 51 suppl 1 90 91 20331728 10.1111/j.1528-1167.2009.02458.x

[3] Weiner HL Carlson C Ridgway EB Epilepsy surgery in young children with tuberous sclerosis: results of a novel approach Pediatrics 2006 117 5 1494 1502 16651302 10.1542/peds.2005-1206

[4] Bebin EM Kelly PJ Gomez MR Surgical treatment for epilepsy in cerebral tuberous sclerosis Epilepsia 1993 34 4 651 657 8330575 10.1111/j.1528-1157.1993.tb00442.x

[5] Bauman JA Feoli E Romanelli P Doyle WK Devinsky O Weiner HL Multistage epilepsy surgery: safety, efficacy, and utility of a novel approach in pediatric extratemporal epilepsy Neurosurgery 2005 56 2 318 334 15670380 10.1227/01.neu.0000148908.71296.fa

[6] Ravindra VM Karas PJ Lazaro TT Epilepsy surgery in young children with tuberous sclerosis complex: a novel hybrid multimodal surgical approach Neurosurgery 2023 92 2 398 406 36637274 10.1227/neu.0000000000002214

[7] Evans LT Morse R Roberts DW Epilepsy surgery in tuberous sclerosis: a review Neurosurg Focus 2012 32 3 E5 10.3171/2012.1.FOCUS11330 22380859

[8] Tovar-Spinoza Z Ziechmann R Zyck S Single and staged laser interstitial thermal therapy ablation for cortical tubers causing refractory epilepsy in pediatric patients Neurosurg Focus 2018 45 3 E9 10.3171/2018.6.FOCUS18228 30173608

